# MOF-Derived Cu@Cu_2_O Nanocatalyst for Oxygen Reduction Reaction and Cycloaddition Reaction

**DOI:** 10.3390/nano8030138

**Published:** 2018-02-28

**Authors:** Aram Kim, Nallal Muthuchamy, Chohye Yoon, Sang Hoon Joo, Kang Hyun Park

**Affiliations:** 1 Department of Chemistry, Pusan National University, Busan 46241, Korea; aramchemi@gmail.com (A.K.); muthuchamyn89@gmail.com (N.M.); yoonch@chemistry.or.kr (C.Y.); 2 School of Energy and Chemical Engineering, Ulsan National Institute of Science and Technology (UNIST), 50 UNIST-gil, Ulsan 44919, Korea; shjoo@unist.ac.kr

**Keywords:** Cu@Cu_2_O, metal-organic framework, nanocatalyst, electrocatalyst, oxygen reduction reaction, Huisgen cycloaddition

## Abstract

Research on the synthesis of nanomaterials using metal-organic frameworks (MOFs), which are characterized by multi-functionality and porosity, as precursors have been accomplished through various synthetic approaches. In this study, copper and copper oxide nanoparticles were fabricated within 30 min by a simple and rapid method involving the reduction of a copper(II)-containing MOF with sodium borohydride solution at room temperature. The obtained nanoparticles consist of a copper core and a copper oxide shell exhibited catalytic activity in the oxygen reduction reaction. The as-synthesized Cu@Cu_2_O core-shell nanocatalyst exhibited an enhanced limit current density as well as onset potential in the electrocatalytic oxygen reduction reaction (ORR). Moreover, the nanoparticles exhibited good catalytic activity in the Huisgen cycloaddition of various substituted azides and alkynes under mild reaction conditions.

## 1. Introduction

The extensive progress in synthesizing and manipulating nano-sized materials made in recent years has led to the development of numerous metal and metal oxide-based nanomaterials. These nanomaterials have been applied in many fields based on their unique properties, including their physical, chemical and electrical stability. In particular, copper and copper oxide-based materials with various oxidation states have received much attention for application in semiconductors, catalysts, energy storage, electrodes, gas sensors and antibiotics [1,2,3,4,5,6,7,8,9,10,11,12,13,14,15,16,17,18]. The catalytic properties of copper and copper oxide-based materials are greatly influenced by the particle size and morphology. Interestingly, copper nanoparticles, in which copper oxide (CuO, Cu_2_O) shells are naturally formed, have different Cu^0^/Cu^+^ surface ratios depending on the particle size and shape. This ratio was found to be an important factor affecting the catalytic activity [19,20]. Such copper-copper oxide (Cu-CuO, Cu-Cu_2_O, Cu_2_O-CuO) core-shell nanoparticles are widely used as catalysts for several organic transformations, including cross-coupling reactions, cycloaddition reactions, catechol oxidation reactions, styrene oxidation reactions, CO oxidation, photocatalytic degradation and reductive degradation [21,22,23,24,25,26,27,28,29,30,31]. In addition, when combined with other metal species or supports, copper/copper oxide hybrid nanoparticles not only exhibit better catalytic activity, but also present superior stability and physicochemical properties. For example, CuFe_2_O_4_ nanoparticles and Fe_3_O_4_@mSiO_2_@Cu_4_ core-shell nanomaterials have the advantage of being magnetically recoverable and reusable, as demonstrated in the N-arylation reaction and oxidation of cycloalkanes [32,33,34]. Co_3_O_4_@CuO, TiO_2_@CuO, Cu/TiO_2_, ZnO-CuO core-branch, Pd/Cu and CuPd-graphene bimetallic nanoparticles exhibit bifunctional catalytic activity and stability for organic reactions and electrocatalysis [35,36,37,38,39].

Recently, metal-organic frameworks (MOFs), comprised of metal species and organic ligands, have attracted attention in the fields of catalysts, sensors, and drug delivery due to their large surface area, multi-functionality and adjustable size, shape and porosity [30,40,41,42,43,44,45,46,47]. In particular, the integration of metal nanoparticles and MOFs has led to enhancement of the physicochemical, magnetic and optical properties of these species. MOFs themselves can be used as supports for encapsulating nanoparticles, and can also be used as precursors for porous graphitic carbon and as shell materials after thermal or chemical treatment of their organic ligands. Moreover, the metal species of MOFs have been employed as precursors to form nanocomposites with uniform shapes and high porosity. By directly producing or incorporating metal nanoparticles into the pores, cavities and channels of MOFs, improved structural properties of the nanomaterials can be achieved, thereby enhancing their application as catalysts, gas sensors and storage materials. In a different approach, nanoparticles have been coated with MOFs to hinder agglomeration of the nanoparticles, thereby retaining the catalytic activity and improving the reusability of the nanoparticles. Furthermore, the metal ions and organic ligands of MOFs can be used as a template or a metal precursor to form a porous metal nanoframe or to generate metal and metal oxide nanoparticles. In the structure of MOF, metal ions combined with organic ligands are scattered throughout the MOF and these scattered metal ions can be reduced to form a uniform sized and shaped nanoparticles without any surfactants and stabilizing agents [47,48,49,50,51,52,53,54].

In this context, we report the generation of MOF-derived Cu@Cu_2_O core-shell nanocatalyst by a facile treatment. The Cu(II)-MOF, formed by the coordination of copper ions and benzene-1,3,5-tricarboxyate linkers, is transformed into smaller Cu@Cu_2_O nanoparticles using sodium borohydride solution as a reducing agent at room temperature. The catalytic activity of the obtained nanoparticles consisting of a copper core and a copper oxide shell, Cu@Cu_2_O core-shell nanocatalyst, in the oxygen reduction reaction and the azide-alkyne Huisgen cycloaddition is also investigated.

## 2. Results and Discussion

### 2.1. Catalyst Characterization

**Figure 1:**
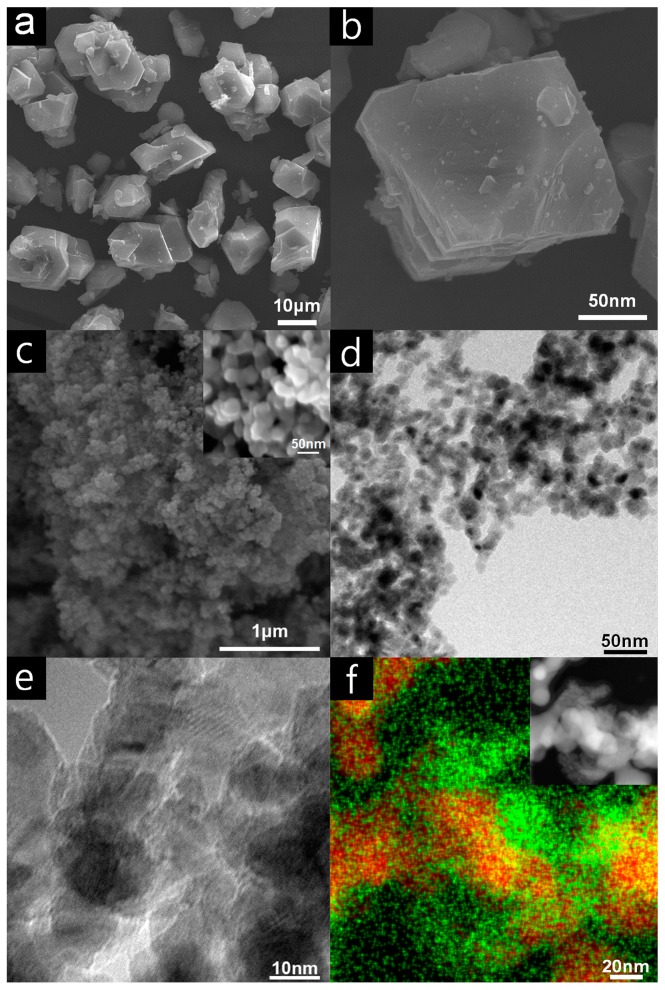
(**a**,**b**) SEM images of Cu(II)-MOF; (**c**) SEM and FE-SEM (inset) images of Cu@Cu_2_O core-shell nanocatalyst and (**d**,**e**) TEM images of Cu@Cu_2_O core-shell nanocatalyst; (**f**) elemental mapping and HAADF-STEM image (inset) of Cu@Cu_2_O core-shell nanocatalyst (red for copper, green for oxygen).

The copper-based MOF, Cu_3_(BTC)_2_ (referred to hereinafter as “Cu(II)-MOF”), was prepared using copper(II) nitrate and H_3_BTC (H_3_BTC = benzene-1,3,5-tricarboxylic acid) in a mixture of *N*,*N*-dimethylformamide, ethanol and water through the solvothermal method reported in the literature [55,56,57,58]. The copper-based MOF was transformed into smaller copper nanoparticles (referred to hereinafter as “Cu@Cu_2_O core-shell nanocatalyst”) by reduction with 10 eq. of sodium borohydride solution. The scanning electron microscope (SEM) images of the Cu(II)-MOF and MOF-derived Cu@Cu_2_O core-shell nanocatalyst are displayed in Figure 1a,b. The Cu(II)-MOF comprised irregular microstructures with an average diameter of approximately 5.0 μm. Generally, the metal nanoparticles which are formed from metal ions of MOF are very close to each other due to the structure of the MOF, so that the metal nanoparticles easily form small agglomerates [16,48,52,59,60]. As shown in Figure 1c–e, the SEM and transmission electron microscope (TEM) images confirm that the copper ions in the Cu(II)-MOF are transformed to spherical nanoparticles with average diameter of approximately 14.8 nm and copper nanoparticles tend to form small agglomerates. The elemental mapping image in Figure 1f shows that more oxygen is distributed on the outer surface of copper nanoparticles showing that surface of the small agglomerate of the copper nanoparticles is unevenly oxidized to copper oxide shell.

**Figure 2:**
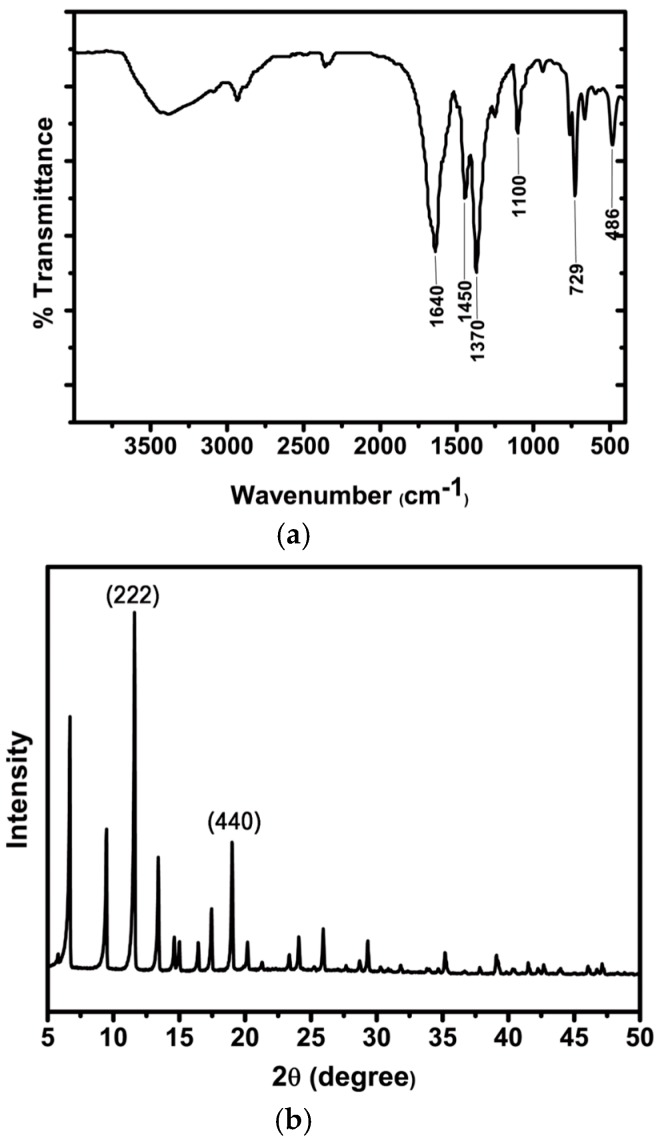

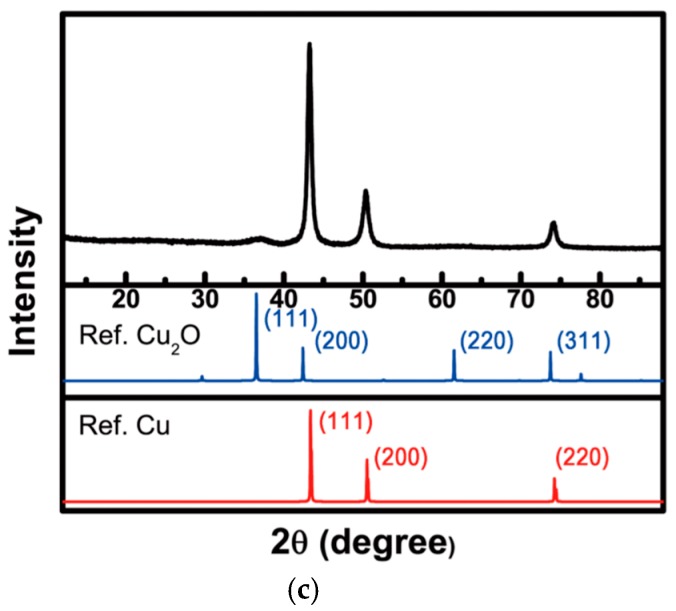
(**a**) FT-IR spectrum of Cu(II)-MOF; X-ray diffraction patterns of (**b**) Cu(II)-MOF and (**c**) Cu@Cu_2_O core-shell nanocatalyst.

The Fourier transform infrared (FT-IR) spectrum and powder X-ray diffraction (XRD) pattern were acquired to verify the formation of Cu(II)-MOF and Cu@Cu_2_O. The FT-IR spectrum of Cu(II)-MOF in Figure 2a shows absorption peaks near 480 and 730 cm^−1^ that can be assigned to the characteristic Cu–O stretching vibration. The characteristic peaks near 1110, 1380 and 1640 cm^−1^ are attributed to CO–Cu stretching and the two CO stretching vibrations of the carboxyl group. The XRD pattern in Figure 2b shows very sharp diffraction peaks at 11.56°, 13.39°, 14.64°, 15.01°, 16.42°, 17.44°, and 19.04°, corresponding to the (222), (400), (331), (420), (422), (333), and (440) crystalline planes of Cu(II)-MOF. Figure 2c shows that the XRD pattern of Cu@Cu_2_O core-shell nanocatalyst is consistent with the reference data for Cu and Cu_2_O. The crystalline size and shell thickness of Cu (111) and Cu_2_O (111) calculated from XRD data are 13.5 nm and 5.07 nm, respectively. And the Cu/Cu_2_O ratio was calculated from ICP and elemental analysis data and the ratio was confirmed as 1:0.8. The presence of copper and oxygen as the main elements was confirmed by ICP and elemental analysis data, and the Cu/Cu_2_O ratio was 1:0.8 which was calculated based on the amount of oxygen.

**Figure 3:**
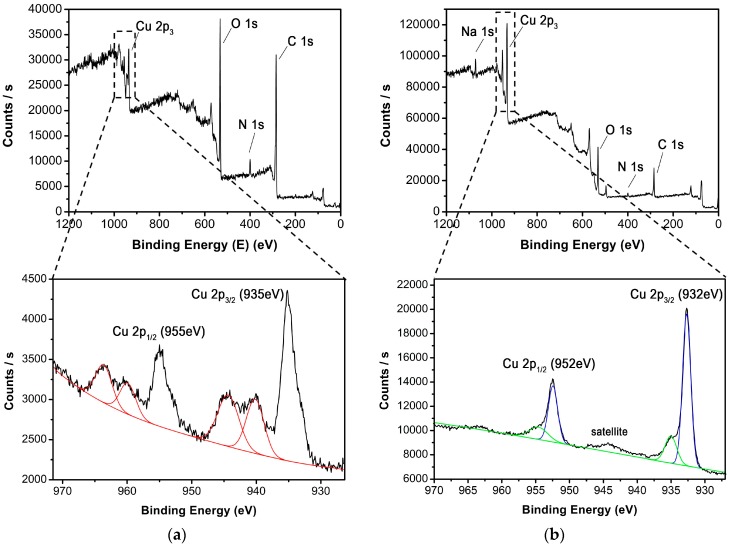
XPS spectra (survey and Cu 2p region) of (**a**) Cu(II)-MOF and (**b**) Cu@Cu_2_O core-shell nanocatalyst.

Furthermore, X-ray photoelectron spectroscopy (XPS) was used to characterize the composition and chemical state of Cu(II)-MOF and Cu@Cu_2_O core-shell nanocatalyst. The XPS survey spectrum of Cu(II)-MOF in Figure 3a shows peaks at ca. 531.8 eV for O 1s, ca. 399.4 eV for N 1s and ca. 284.6 eV for C 1s; two characteristic peaks of Cu(II) were observed in the Cu 2p region at binding energies of 934.9 and 954.7 eV, corresponding to Cu 2p_3/2_ and Cu 2p1_/2_, respectively. Furthermore, Cu(II)-MOF (Figure 3a) reveals the presence of two strong satellite features on the higher binding energies centered at 938–946 eV and 961–965 eV corresponding to Cu 2p, which is also evidence the presence of Cu(II) in Cu(II)-MOF. To note, the binding energy values of the Cu 2p_3/2_ (932 eV) and Cu 2p_1/2_ (952 eV) in Cu@Cu_2_O core-shell nanocatalyst (Figure 3b) are shifted as compared to the Cu 2p_3/2_ and Cu 2p_1/2_ values in Cu(II)-MOF (Figure 3a). And, the binding energy of peaks corresponding to the satellite features of Cu@Cu_2_O core-shell nanocatalyst (Figure 3b) showed very tiny peak intensities (due to the insignificant amount of CuO formation from slightly oxidized surface of Cu_2_O shell via atmospheric exposure) as compared to the peak intensities of Cu(II)-MOF (Figure 3a). Thus, the shifts in the binding energy values of Cu 2p_3/2_, Cu 2p_1/2_ and reduction of strong satellite (Figure 3a, 938–946 eV and 961–965 eV) to very tiny peak observed at 945 eV suggests the possibilities of formations of Cu and Cu_2_O from Cu(II)-MOF and effective stable nature of Cu_2_O on Cu@Cu_2_O core-shell nanocatalyst [61,62]. Considering both the XRD and XPS results, it is reasonable to deduce that the copper ions in the MOF were adequately reduced to Cu@Cu_2_O core-shell nanocatalyst.

### 2.2. Electrocatalytic Activity of Cu@Cu_2_O Core-Shell Nanocatalyst

**Figure 4:**
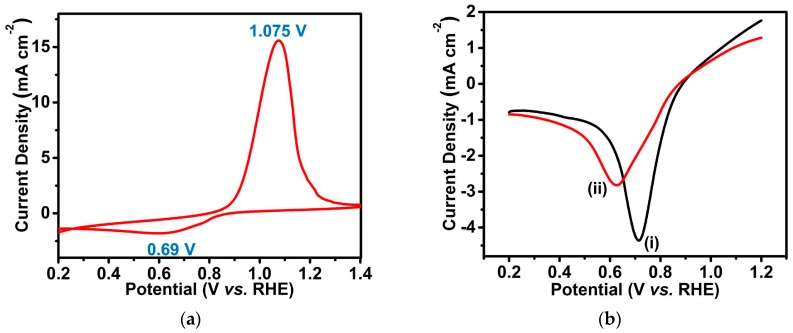
(**a**) CV curve of Cu@Cu_2_O core-shell nanocatalyst on glassy carbon disk electrode in O_2_-saturated 0.1 M KOH with a scan rate of 0.02 V s^−1^; (**b**) LSV curves of Cu@Cu_2_O core-shell nanocatalyst on glassy carbon electrodes in O_2_-saturated (i) and Ar-saturated (ii) 0.1 M KOH with a scan rate of 0.02 V s^−1^.

In order to assess the ORR electrocatalytic activity of the Cu@Cu_2_O core-shell nanocatalyst, cyclic voltammetry (CV) and linear sweep voltammetry (LSV) in O_2_-saturated 0.1 M KOH using a glass carbon (GC) disk electrode loaded with the Cu@Cu_2_O core-shell nanocatalyst were employed as convenient and efficient tools (as described in the Materials and Methods section). The CV curve of the Cu@Cu_2_O core-shell nanocatalyst (Figure 4a) was used to explore the redox reactions involved. In the strong 0.1 M KOH electrolyte, a pair of anodic (1.075 V vs. reversible hydrogen electrode (RHE)) and cathodic (0.69 V vs. RHE) peaks was clearly observed at a scan rate of 0.02 V s^−1^, which indicates Faradaic redox reactions (Cu^2+^/Cu^+^) of the Cu@Cu_2_O core-shell nanocatalyst. Further, the electrocatalytic activity towards the ORR is clearly demonstrated in Figure 4b, where the oxygen reduction peak potential shifted to a more positive direction with a high peak current density in the presence of O_2_ (Figure 4b curve i) than in the presence of Ar (Figure 4b curve ii).

**Figure 5:**
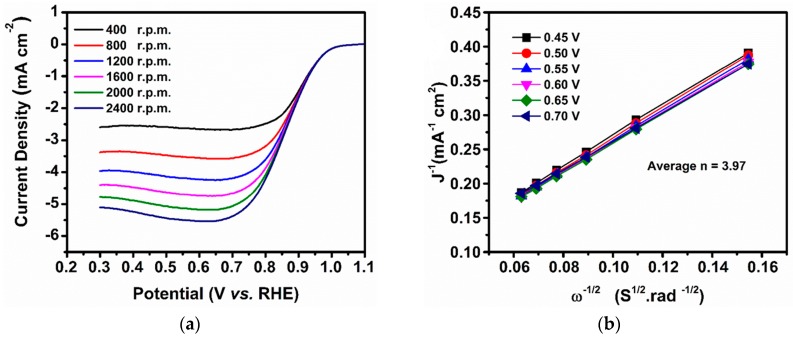
(**a**) Steady-state voltammograms of the ORR profiles at different rotation rates (400–2400 r.p.m.) and (**b**) Koutecky–Levich (K–L) plots of Cu@Cu_2_O core-shell nanocatalyst in O_2_-saturated 0.1 M KOH solution with a scan rate of 0.02 V s^−1^.

In order to elucidate the mechanism of enhancement of the electrocatalytic activity of the Cu@Cu_2_O core-shell nanocatalyst in the ORR, LSV measurements (Figure 5 a) were performed using a rotating-disk electrode (RDE) in the applied potential range of 0.2–1.4 V vs. RHE in O_2_-saturated 0.1 M KOH solution (scan rate = 0.02 V s^−1^; the electrode rotating speed was varied as 400, 800, 1200, 1600, 2000 and 2500 r.p.m.). The ORR polarization curves of the Cu@Cu_2_O core-shell nanocatalyst demonstrate enhancement of the limit current density as well as the more positive onset potential (*E_onset_* = 0.93 V vs. RHE). The Cu@Cu_2_O core-shell nanocatalyst showed a half-wave potential (*E*_1/2_) of 0.86 V vs. RHE at 1600 r.p.m., which is comparatively higher than the reported values for Cu-based ORR catalysts such as rGO–TADPyCu (*E*_1/2_ = 0.795 V) [63], Cu-N_x_/C [0.755 V] [64]. To quantitatively evaluate the ORR electrocatalytic activity of Cu@Cu_2_O core-shell nanocatalyst, the Koutecky–Levich (K–L) plots based on the LSV measurements in the potential range of 0.45–0.70 V vs. RHE at various rotating speeds were used to calculate the corresponding electron transfer number (Figure 5b). The corresponding K–L plots show good linearity and also parallelism of plots indicate the first order kinetics towards ORR in alkaline electrolyte. The electron transfer number of Cu@Cu_2_O core-shell nanocatalyst estimated from the slope of the K–L plots is averagely 3.97 at 0.45–0.7 V, suggested a principal 4-electron transfer pathway. The *n* = 3.97 is higher than already reported on various shape controlled Cu_2_O nanostructures (Cu_2_O-70 (*n* = 3.74); Cu_2_O-50 (*n* = 3.22) and Cu_2_O-20 (*n* = 2.04)) [65]. These are confirmed that the Cu@Cu_2_O core-shell nanocatalyst has direct 4-electron transfer pathway in ORR due to the synergistic morphological effects of a core-shell nanostructure, well dispersed Cu_2_O and the high, strong adsorption of O_2_ on Cu@Cu_2_O surfaces. There are might be the reasons for the superior electrocatalytic activity of Cu@Cu_2_O core-shell nanocatalyst towards ORR. These results evidently suggested that the prepared Cu@Cu_2_O core-shell nanocatalyst is the highly selective 4-electron transfer pathway with more positive *E_onset_* = 0.93 V vs. RHE toward ORR.

### 2.3. Catalytic Activity of Cu@Cu_2_O Core-Shell Nanocatalyst in Azide-Alkyne Huisgen Cycloaddition

Huisgen azide-alkyne cycloaddition is a representative reaction of click chemistry, especially under the copper catalysis, produces a 1,2,3-triazoles as a product which can be applied in organic synthesis, pharmaceuticals and biological materials [66,67,68,69,70,71]. As shown in Table 1, the catalytic activity of the Cu@Cu_2_O core-shell nanocatalyst was evaluated in the Huisgen cycloaddition of benzyl azide to phenyl acetylene. The catalytic reactions were carried out at 50 °C for 5 h using benzyl azide, phenylacetylene and 2.3 mol % of catalyst. The reaction performed with the Cu@Cu_2_O core-shell nanocatalyst led to complete conversion to the 1,4-disubstituted 1,2,3-triazoles as the desired products, whereas the reaction carried out using Cu(II)-MOF as the catalyst resulted in a low conversion of 12%. Moreover, it was found that the cycloaddition reactions with electron-withdrawing substituents and electron-donating substituents were efficiently catalyzed by the Cu@Cu_2_O core-shell nanocatalyst. As examples, the hydroxy-substituted alkynes gave the expected products, i.e., (1-benzyltriazol-4-yl)methanol and 2-(1-benzyl-1H-1,2,3-triazol-4-yl)propan-2-ol, as single regioisomers with complete conversion (Table 1, 1d and 1e). The ortho- and para-methoxy group respectively gave the expected 1-(4-methoxyphenyl)-4-phenyl-1H-1,2,3-triazole and 1-(2-methoxyphenyl)-4-phenyl-1H-1,2,3-triazole as a single regioisomer with 99% and 87% conversion (Table 1, 1j and 1k). In a recent related study, copper-catalyzed Huisgen azide-alkyne cycloadditions were conducted at room temperature to 70 °C and reaction time 3–12 h using water and alcohol as solvent to achieve high yield. The prepared Cu@Cu_2_O core-shell nanocatalyst provided the desired triazole with satisfactory conversion under similar mild reaction conditions [72,73,74,75,76].

## 3. Materials and Methods

### 3.1. General Remarks

The morphology of the samples was analyzed by using a Zeiss Supra 40 VP field emission scanning electron microscope (FE-SEM) and FEI Quanta 200 scanning electron microscope (FEI, Hillsboro, OR, USA) operating at 15 kV. The size and morphology were characterized by using a JEOL JEM-2100F transmission electron microscope (JEOL Ltd., Tokyo, Japan) at an accelerating voltage of 200 kV. X-ray diffraction (XRD) patterns were recorded on a Rigaku D/MAX-RB (12 kW; Rigaku, Shibuya-ku, Japan) diffractometer. Fourier-transform infrared spectra (FT-IR) and X-ray photoelectron spectra (XPS) were recorded on Nicolet 380 (Thermo, Waltham, MA, USA) and ESCALab250 (Thermo, Waltham, MA, USA) instruments, respectively. Electrochemical measurements were performed by using an electrochemical workstation (CHI600E, CH Instruments, Austin, TX, USA) with a three-electrode system. The progress of the catalytic reaction was observed by gas chromatography-mass spectrometry (GC/MS; Shimadzu-QP2010 SE, Shimadzu, Kyoto, Japan). All chemicals were used as received without further purification.

### 3.2. Synthesis of Cu_3_(BTC)_2_ MOF (Cu(II)-MOF) and Cu@Cu_2_O Core-Shell Nanocatalyst

Cu(NO_3_)_2_∙2.5H_2_O (10 g) and benzene-1,3,5-tricarboxylic acid (H_3_BTC; 5 g) were combined in 250 mL of a mixture of dimethyl formamide (DMF), ethanol and water (1:1:1 *v*/*v*). The mixture was stirred for 10 min and heated to reflux at 85 °C for 20 h. The obtained blue crystals were washed and filtered with DMF and then dried at 60 °C for 5 h.

The as-synthesized Cu(II)-MOF (0.5 g) was dissolved in deionized (DI) water (20 mL) with stirring. NaBH_4_ solution (10 eq.) was added dropwise with stirring in an ice-bath. The mixture was stirred at room temperature for 30 min. The resultant particles were washed with water and ethanol and dried under vacuum.

### 3.3. Electrode Preparation and Electrochemical Measurements

The electrochemical and electrocatalytic performance of the Cu@Cu_2_O core-shell nanocatalyst was investigated through cyclic voltammetry (CV) and by using a rotating disc electrode (RDE, AFMSRCE, Pine Research Instruments, Durham, NC, USA) in an O_2_-saturated (0.1 M KOH) electrolyte at a scan rate of 20 mV s^−1^. For the three-electrode system, a silver/silver chloride (Ag/AgCl) and graphite electrode were used as the reference and counter electrodes, respectively. The Cu@Cu_2_O core-shell nanocatalyst (5 mg) was dispersed in 200 µL of ink solution containing isopropyl alcohol (140 µL), deionized water (40 µL) and Nafion solution (20 µL) in a 7:2:1 (*v*/*v*) ratio and the mixture was ultrasonicated for 1 h to obtain a homogeneous suspension of the prepared ink solution. The Cu@Cu_2_O core-shell nanocatalyst ink (10 μL) was dropped onto the surface of a glassy carbon disk (working electrode, area = 0.1963 cm^2^) and dried at 60 °C for 30 min. A conventional RDE three-compartmental glass cell was used. All experiments were performed at room temperature. The ORR performance was measured in O_2_-saturated 0.1 M KOH electrolyte. To avoid the influence of the reduction of Cu^2+^ and set the background correction, an Ar-saturated 0.1 M KOH electrolyte was used under the same experimental conditions. In this study, all reported potentials were converted from the Ag/AgCl to the RHE scale using *E* (RHE) = *E* (Ag/AgCl) + 0.198 V in 0.1 M KOH. The number of electrons transferred during the ORR was calculated from the Koutecky–Levich (K–L) plot, which is determined by Equations (1) and (2).

(1) $$1/J=1/J_{L}+1/J_{K}=1/B\omega^{1/2}+1/J_{K}$$

where *J*, *J_L_* and *J_K_* are the values of the experimentally measured current and kinetic and diffusion-limiting current densities, respectively; *ω* is the angular velocity of the disk, and *B* can be defined as follows:

(2) $$B=0.62nFC_{O}{\left(D_{O}\right)}^{2/3}\vartheta^{-1/6}$$

where *F* is the Faraday constant (96,485.34 C mol^−1^), *C_O_* is the bulk concentration of O_2_ (1.2 × 10^−6^ mol cm^−3^), *ϑ* is the kinematic viscosity of the electrolyte (*ϑ* = 0.01 cm^2^ s^−1^, *D_O_* is the diffusion coefficient of O_2_ in 0.1 M KOH (1.9 × 10^−5^ cm^2^ s^−1^) and *n* is the number of electrons transferred during the electrochemical reaction. The value of *n* can be calculated from the slope of the *J*^−1^ vs. *ω*^−1⁄2^ plot.

### 3.4. General Procedure for Azide-Alkyne Huisgen Cycloadditions

Benzyl azide (1 mmol), phenyl acetylene (1.5 mmol), water (2.0 mL), tert-butyl alcohol (1.0 mL) and Cu@Cu_2_O nanocatalyst (2.3 mol %) were placed into a 10 mL sealed aluminum vial with a butyl gum septum. The mixture was stirred at 50 °C for 5 h. After the reaction, the Cu@Cu_2_O core-shell nanocatalyst were separated from the clear solution. The products were analyzed by ^1^H-NMR and GC/MS.

## 4. Conclusions

In summary, Cu@Cu_2_O core-shell nanocatalyst with a diameter of 74 nm was easily synthesized through the sodium borohydride reduction of Cu(II)-MOF and Cu_3_(BTC)_2_. The ORR polarization curves of the Cu@Cu_2_O core-shell nanocatalyst show an improved limit current density and onset potential (*E_onset_* = 0.93 V vs. RHE). The electron transfer number for the Cu@Cu_2_O core-shell nanocatalyst is 3.97 and this catalyst operates by a 4-electron transfer pathway in the ORR. We have reason to believe that the Cu@Cu_2_O core-shell nanocatalyst will extend the understanding of non-noble metal/metal oxide catalysis and direct the rational strategy of non-noble metal/metal oxide catalysts for the ORR. Also, Cu@Cu_2_O core-shell nanocatalyst exhibits good catalytic activity in the azide-alkyne Huisgen cycloaddition of various substituents under mild reaction conditions. The simply-obtained MOF-derived copper-copper oxide nanoparticles were successfully employed as a nanocatalyst in both organic reactions and electrochemical catalysis. The prepared Cu@Cu_2_O core-shell nanocatalyst can circumvent the problems encountered with peroxide, such as corrosion or premature degradation of the cells. However, in order to replace costly platinum-based catalysts in fuel cells with the current type of composites, systematic studies should be conducted to enhance the kinetic current density as well as stability of the catalysts.

## Figures and Tables

**Table 1:** Huisgen cycloaddition of azides with terminal alkynes catalyzed by Cu@Cu_2_O core-shell nanocatalyst ^1^.

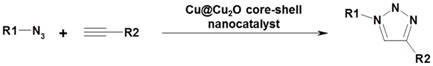		
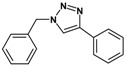 1a, 100%	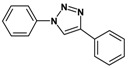 1b, 95%	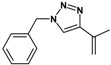 1c, 80%
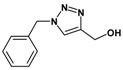 1d, 100%	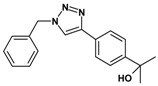 1e, 100%	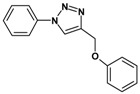 1f, 64%
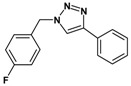 1g, 69%	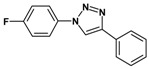 1h, 83%	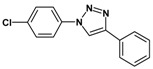 1i, 97%
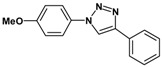 1j, 99%	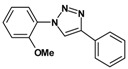 1k, 87%	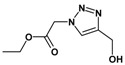 1l, 100%

^1^ Reaction conditions: azide (1.0 mmol), alkyne (1.5 mmol), Cu@Cu_2_O core-shell nanocatalyst (2.3 mol %), water:t-BuOH = 2:1, 50 °C, 5 h.

## References

[1] Wang H., Zhu Q.L., Zou R., Xu Q. (2017). Metal-organic frameworks for energy applications. Chem.

[2] Woo H., Kim E., Kim J.H., Yun S.W., Park J.C., Kim Y.T., Park K.H. (2017). Shape and Composition Control of Monodisperse Hybrid Pt-CoO Nanocrystals by Controlling the Reaction Kinetics with Additives. Sci. Rep..

[3] Kim A., Shin D., Kim M., Yoon C., Song H., Park K.H. (2014). Facile synthesis of multipodal MnO nanocrystals and their catalytic performance. Eur. J. Inorg. Chem..

[4] Komathi S., Gopalan A.I., Muthuchamy N., Lee K.P. (2017). Polyaniline nanoflowers grafted onto nanodiamonds via a soft template-guided secondary nucleation process for high-performance glucose sensing. RSC Adv..

[5] Jang S., Sa Y.J., Joo S.H., Park K.H. (2016). Ordered mesoporous copper oxide nanostructures as highly active and stable catalysts for aqueous click reactions. Catal. Commun..

[6] Gopalan A.I., Muthuchamy N., Lee K.P. (2017). A novel bismuth oxychloride-graphene hybrid nanosheets based non-enzymatic photoelectrochemical glucose sensing platform for high performances. Biosens. Bioelectron..

[7] Deutsch M., Horvath F., Knoll C., Lager D., Gierl-Mayer C., Weinberger P., Winter F. (2017). High-temperature energy storage: Kinetic investigations of the CuO/Cu_2_O reaction cycle. Energy Fuels.

[8] Park H.J., Choi N.J., Kang H., Jung M.Y., Park J.W., Park K.H., Lee D.S. (2014). A ppb-level formaldehyde gas sensor based on CuO nanocubes prepared using a polyol process. Sens. Actuators B Chem..

[9] Zhan J., Chen M., Xia X. (2015). Controllable synthesis of copper oxide/carbon core/shell nanowire arrays and their application for electrochemical energy storage. Nanomaterials.

[10] Muthuchamy N., Gopalan A.I., Lee K.P. (2017). New titanium dioxide-based heterojunction nanohybrid for highly selective photoelectrochemical–electrochemical dual-mode sensors. ACS Appl. Mater. Interfaces.

[11] Zhou L., He Y., Jia C., Pavlinek V., Saha P., Cheng Q. (2017). Construction of hierarchical CuO/Cu_2_O@NiCo_2_S_4_ nanowire arrays on copper foam for high performance supercapacitor electrodes. Nanomaterials.

[12] Li X., Li X., Chen N., Li X., Zhang J., Yu J., Wang J., Tang Z. (2014). CuO-In_2_O_3_ core-shell nanowire based chemical gas sensors. J. Nanomater..

[13] Muthuchamy N., Lee K.P., Gopalan A.I. (2017). Enhanced photoelectrochemical biosensing performances for graphene (2D)–Titanium dioxide nanowire (1D) heterojunction polymer conductive nanosponges. Biosens. Bioelectron..

[14] Muthuchamy N., Gopalan A., Lee K.P. (2015). A new facile strategy for higher loading of silver nanoparticles onto silica for efficient catalytic reduction of 4-nitrophenol. RSC Adv..

[15] Ahamed M., Alhadlaq H.A., Khan M., Karuppiah P., Al-Dhabi N.A. (2014). Synthesis, characterization, and antimicrobial activity of copper oxide nanoparticles. J. Nanomater..

[16] Liu B., Zhang X., Shioyama H., Mukai T., Sakai T., Xu Q. (2010). Converting cobalt oxide subunits in cobalt metal-organic framework into agglomerated Co_3_O_4_ nanoparticles as an electrode material for lithium ion battery. J. Power Sources.

[17] Muthuchamy N., Gopalan A.I., Lee K.P. (2018). Highly selective non-enzymatic electrochemical sensor based on a titanium dioxide nanowire–poly(3-aminophenyl boronic acid)–gold nanoparticle ternary nanocomposite. RSC Adv..

[18] Hsueh Y.-H., Tsai P.-H., Lin K.-S. (2017). Ph-dependent antimicrobial properties of copper oxide nanoparticles in staphylococcus aureus. Int. J. Mol. Sci..

[19] Konar S., Kalita H., Puvvada N., Tantubay S., Mahto M.K., Biswas S., Pathak A. (2016). Shape-dependent catalytic activity of CuO nanostructures. J. Catal..

[20] Caldas P.C., Gallo J.M.R., Lopez-Castillo A., Zanchet D., Correa Bueno J.M. (2017). The structure of the Cu–CuO sites determines the catalytic activity of cu nanoparticles. ACS Catal..

[21] Pan K., Ming H., Yu H., Liu Y., Kang Z., Zhang H., Lee S.T. (2011). Different copper oxide nanostructures: Synthesis, characterization, and application for C-N cross-coupling catalysis. Cryst. Res. Technol..

[22] Ranu B.C., Dey R., Chatterjee T., Ahammed S. (2012). Copper nanoparticle-catalyzed carbon-carbon and carbon-heteroatom bond formation with a greener perspective. ChemSusChem.

[23] Amini M., Pourvahabi Anbari A., Ramezani S., Gautam S., Hwa Chae K. (2016). Copper(II) oxide nanoparticles as an efficient catalyst in the azide–alkynecycloaddition. ChemistrySelect.

[24] Woo H., Kang H., Kim A., Jang S., Park J.C., Park S., Kim B.-S., Song H., Park K.H. (2012). Azide-alkyne Huisgen [3 + 2] cycloaddition using CuO nanoparticles. Molecules.

[25] Kang H., Lee H.J., Park J.C., Song H., Park K.H. (2010). Solvent-free microwave promoted [3 + 2] cycloaddition of alkyne-azide in uniform CuO hollow nanospheres. Top. Catal..

[26] Kim A., Sharma B., Kim B.-S., Park K.H. (2011). Double-hydrophilic block copolymer nanoreactor for the synthesis of copper nanoparticles and for application in click chemistry. J. Nanosci. Nanotechnol..

[27] Jang S., Yoon C., Lee J.M., Park S., Park K.H. (2016). Preparation of Cu@Cu_2_O nanocatalysts by reduction of HKUST-1 for oxidation reaction of catechol. Molecules.

[28] Mu H., Li C., Bai J. (2017). The composite catalysts of Cu/Cu_x_O nanoparticles supported on the carbon fibers were prepared for styrene oxidation reaction. Appl. Organomet. Chem..

[29] Gonçalves R.V., Wojcieszak R., Wender H., Sato B., Dias C., Vono L.L., Eberhardt D., Teixeira S.R., Rossi L.M. (2015). Easy access to metallic copper nanoparticles with high activity and stability for co oxidation. ACS Appl. Mater. Interfaces.

[30] Xu L., Srinivasakannan C., Peng J., Zhang L., Zhang D. (2017). Synthesis of Cu-CuO nanocomposite in microreactor and its application to photocatalytic degradation. J. Alloys Compd..

[31] Zhao X., Tan Y., Wu F., Niu H., Tang Z., Cai Y., Giesy J.P. (2016). Cu/Cu_2_O/CuO loaded on the carbon layer derived from novel precursors with amazing catalytic performance. Sci. Total Environ..

[32] Panda N., Jena A.K., Mohapatra S., Rout S.R. (2011). Copper ferrite nanoparticle-mediated N-arylation of heterocycles: A ligand-free reaction. Tetrahedron Lett..

[33] Yang D., Zhu X., Wei W., Sun N., Yuan L., Jiang M., You J., Wang H. (2014). Magnetically recoverable and reusable CuFe_2_O_4_ nanoparticle-catalyzed synthesis of benzoxazoles, benzothiazoles and benzimidazoles using dioxygen as oxidant. RSC Adv..

[34] Kirillova M.V., Santos C.I., Wu W., Tang Y., Kirillov A.M. (2017). Mild oxidative C−H functionalization of alkanes and alcohols using a magnetic core-shell Fe_3_O_4_@_2_@Cu_4_ nanocatalyst. J. Mol. Catal. A Chem..

[35] Yang W., Salim J., Ma C., Ma Z., Sun C., Li J., Chen L., Kim Y. (2013). Flowerlike Co_3_O_4_ microspheres loaded with copper nanoparticle as an efficient bifunctional catalyst for lithium–air batteries. Electrochem. Commun..

[36] Kaur R., Pal B. (2015). Cu nanostructures of various shapes and sizes as superior catalysts for nitro-aromatic reduction and co-catalyst for Cu/TiO_2_ photocatalysis. Appl. Catal. A Gen..

[37] Gopalan A.I., Muthuchamy N., Komathi S., Lee K.P. (2016). A novel multicomponent redox polymer nanobead based high performance non-enzymatic glucose sensor. Biosens. Bioelectron..

[38] Dong Q., Zhao Y., Han X., Wang Y., Liu M., Li Y. (2014). Pd/Cu bimetallic nanoparticles supported on graphene nanosheets: Facile synthesis and application as novel electrocatalyst for ethanol oxidation in alkaline media. Int. J. Hydrog. Energy.

[39] Jin X., Dang L., Lohrman J., Subramaniam B., Ren S., Chaudhari R.V. (2013). Lattice-matched bimetallic CuPd-graphene nanocatalysts for facile conversion of biomass-derived polyols to chemicals. ACS Nano.

[40] Liu Y., Tang Z. (2013). Multifunctional nanoparticle@MOF core–shell nanostructures. Adv. Mater..

[41] Dang S., Zhu Q.L., Xu Q. (2017). Nanomaterials derived from metal–organic frameworks. Nat. Rev. Mater..

[42] Yang J.-M., Liu Q., Sun W.-Y. (2014). Co(II)-doped MOF-5 nano/microcrystals: Solvatochromic behaviour, sensing solvent molecules and gas sorption property. J. Solid State Chem..

[43] Wan M., Zhang X., Li M., Chen B., Yin J., Jin H., Lin L., Chen C., Zhang N. (2017). Hollow Pd/MOF nanosphere with double shells as multifunctional catalyst for hydrogenation reaction. Small.

[44] Sava Gallis D.F., Rohwer L.E., Rodriguez M.A., Barnhart-Dailey M.C., Butler K.S., Luk T.S., Timlin J.A., Chapman K.W. (2017). Multifunctional, tunable metal–organic framework materials platform for bioimaging applications. ACS Appl. Mater. Interfaces.

[45] Wang X., Zhou J., Fu H., Li W., Fan X., Xin G., Zheng J., Li X. (2014). MOF derived catalysts for electrochemical oxygen reduction. J. Mater. Chem. A.

[46] Ma D., Li B., Liu K., Zhang X., Zou W., Yang Y., Li G., Shi Z., Feng S. (2015). Bifunctional MOF heterogeneous catalysts based on the synergy of dual functional sites for efficient conversion of CO_2_ under mild and co-catalyst free conditions. J. Mater. Chem. A.

[47] Wang T., Zhou Q., Wang X., Zheng J., Li X. (2015). MOF-derived surface modified Ni nanoparticles as an efficient catalyst for the hydrogen evolution reaction. J. Mater. Chem. A.

[48] Meng F., Fang Z., Li Z., Xu W., Wang M., Liu Y., Zhang J., Wang W., Zhao D., Guo X. (2013). Porous Co_3_O_4_ materials prepared by solid-state thermolysis of a novel Co-MOF crystal and their superior energy storage performances for supercapacitors. J. Mater. Chem. A.

[49] Bai C., Li A., Yao X., Liu H., Li Y. (2016). Efficient and selective aerobic oxidation of alcohols catalysed by MOF-derived Co catalysts. Green Chem..

[50] Liu J., Wu C., Xiao D., Kopold P., Gu L., van Aken P.A., Maier J., Yu Y. (2016). MOF-derived gollow Co_9_S_8_ nanoparticles embedded in graphitic carbon nanocages with superior Li-ion storage. Small.

[51] Liu H., Zhang S., Liu Y., Yang Z., Feng X., Lu X., Huo F. (2015). Well-dispersed and size-controlled supported metal oxide nanoparticles derived from MOF composites and further application in catalysis. Small.

[52] Lü Y., Zhan W., He Y., Wang Y., Kong X., Kuang Q., Xie Z., Zheng L. (2014). MOF-templated synthesis of porous Co_3_O_4_ concave nanocubes with high specific surface area and their gas sensing properties. ACS Appl. Mater. Interfaces.

[53] Kundu T., Sahoo S.C., Banerjee R. (2012). Solid-state thermolysis of anion induced metal–organic frameworks to ZnO microparticles with predefined morphologies: Facile synthesis and solar cell studies. Cryst. Growth Des..

[54] Wang Y., Sang S., Zhu W., Gao L., Xiao G. (2016). CuNi@C catalysts with high activity derived from metal–organic frameworks precursor for conversion of furfural to cyclopentanone. Chem. Eng. J..

[55] Rowsell J.L., Yaghi O.M. (2006). Effects of functionalization, catenation, and variation of the metal oxide and organic linking units on the low-pressure hydrogen adsorption properties of metal−organic frameworks. J. Am. Chem. Soc..

[56] Okada K., Ricco R., Tokudome Y., Styles M.J., Hill A.J., Takahashi M., Falcaro P. (2014). Copper conversion into Cu(OH)_2_ nanotubes for positioning Cu_3_(BTC)_2_ MOF crystals: Controlling the growth on flat plates, 3d architectures, and as patterns. Adv. Funct. Mater..

[57] Xiang Z., Cao D., Shao X., Wang W., Zhang J., Wu W. (2010). Facile preparation of high-capacity hydrogen storage metal-organic frameworks: A combination of microwave-assisted solvothermal synthesis and supercritical activation. Chem. Eng. Sci..

[58] Petit C., Burress J., Bandosz T.J. (2011). The synthesis and characterization of copper-based metal–organic framework/graphite oxide composites. Carbon.

[59] Song M.J., Kim I.T., Kim Y.B., Kim J., Shin M.W. (2017). Metal–organic frameworks-derived porous carbon/Co_3_O_4_ composites for rechargeable lithium–oxygen batteries. Electrochimica Acta.

[60] Chen B., Ma G., Zhu Y., Xia Y. (2017). Metal-organic-frameworks derived cobalt embedded in various carbon structures as bifunctional electrocatalysts for oxygen reduction and evolution reactions. Sci. Rep..

[61] Deo M., Mujawar S., Game O., Yengantiwar A., Banpurkar A., Kulkarni S., Jog J., Ogale S. (2011). Strong photo-response in a flip-chip nanowire p-Cu_2_O/n-ZnO junction. Nanoscale.

[62] Poulston S., Parlett P., Stone P., Bowker M. (1996). Surface oxidation and reduction of CuO and Cu_2_O studied using XPS and XAES. Surf. Interface Anal..

[63] Xi Y.-T., Wei P.-J., Wang R.-C., Liu J.-G. (2015). Bio-inspired multinuclear copper complexes covalently immobilized on reduced graphene oxide as efficient electrocatalysts for the oxygen reduction reaction. Chem. Commun..

[64] He Q., Yang X., He R., Bueno-López A., Miller H., Ren X., Yang W., Koel B.E. (2012). Electrochemical and spectroscopic study of novel Cu and Fe-based catalysts for oxygen reduction in alkaline media. J. Power Sources.

[65] Zhang X., Zhang Y., Huang H., Cai J., Ding K., Lin S. (2018). Electrochemical fabrication of shape-controlled Cu_2_O with spheres, octahedrons and truncated octahedrons and their electrocatalysis for ORR. New J. Chem..

[66] Sharpless K.B., Manetsch R. (2006). In situ click chemistry: A powerful means for lead discovery. Expert Opin. Drug Discov..

[67] Moses J.E., Moorhouse A.D. (2007). The growing applications of click chemistry. Chem. Soc. Rev..

[68] Agalave S.G., Maujan S.R., Pore V.S. (2011). Click chemistry: 1,2,3-triazoles as pharmacophores. Chemistry.

[69] Zheng T., Rouhanifard S.H., Jalloh A.S., Wu P. (2012). Click triazoles for bioconjugation. Click Triazoles.

[70] Breinbauer R., Köhn M. (2003). Azide–alkyne coupling: A powerful reaction for bioconjugate chemistry. ChemBioChem.

[71] Xi W., Scott T.F., Kloxin C.J., Bowman C.N. (2014). Click chemistry in materials science. Adv. Funct. Mater..

[72] Amini M., Naslhajian H., Farnia S.M.F., Kang H.K., Gautam S., Chae K.H. (2016). Polyoxomolybdate-stabilized Cu_2_O nanoparticles as an efficient catalyst for the azide–alkyne cycloaddition. New J. Chem..

[73] Faraji M., Amini M., Anbari A.P. (2016). Preparation and characterization of TiO_2_-nanotube/Ti plates loaded Cu_2_O nanoparticles as a novel heterogeneous catalyst for the azide–alkyne cycloaddition. Catal. Commun..

[74] Kimber R.L., Lewis E.A., Parmeggiani F., Smith K., Bagshaw H., Starborg T., Joshi N., Figueroa A.I., van der Laan G., Cibin G. (2018). Biosynthesis and characterization of copper nanoparticles using *Shewanella oneidensis*: Application for click chemistry. Small.

[75] Ghosh S., Saha S., Sengupta D., Chattopadhyay S., De G., Basu B. (2017). Stabilized Cu_2_O nanoparticles on macroporous polystyrene resins [Cu_2_O@ARF]: Improved and reusable heterogeneous catalyst for on-water synthesis of triazoles via click reaction. Ind. Eng. Chem. Res..

[76] Shaabani S., Tavousi Tabatabaei A., Shaabani A. (2017). Copper(I) oxide nanoparticles supported on magnetic casein as a bio-supported and magnetically recoverable catalyst for aqueous click chemistry synthesis of 1,4-disubstituted 1,2,3-triazoles. Appl. Organomet. Chem..

